# Transferability of Simulation-Based Training in Laparoscopic Surgeries: A Systematic Review

**DOI:** 10.1155/2020/5879485

**Published:** 2020-08-25

**Authors:** Antonios E. Spiliotis, Panagiotis M. Spiliotis, Ifaistion M. Palios

**Affiliations:** ^1^Department of General, Visceral, Vascular and Pediatric Surgery, Saarland University, Saarland University Medical Center, Homburg, Germany; ^2^Department of Surgery, University Hospital Knappschaftskrankenhaus Bochum, Ruhr-University Bochum, Bochum, Germany; ^3^Department of Surgery, Laiko General Hospital, National and Kapodistrian University of Athens, Athens, Greece

## Abstract

**Objective:**

The implementation of simulation-based training in residency programs has been increased, but the transferability of surgical skills in the real operating room is not well documented. In our survey, the role of simulation in surgical training will be evaluated. *Study Design*. In this systemic review, randomized control trials, which assessed the transferability of acquired skills through simulation in the real operating setting, were included. A systematic search strategy was undertaken using a predetermined protocol.

**Results:**

Eighteen randomized clinical trials were included in this survey. Two studies investigated inguinal hernia repair, six laparoscopic cholecystectomy, five gynecologic procedures, two laparoscopic suturing, and two camera navigation during laparoscopic procedures. Simulation-trained participants showed superiority in surgical performance in comparison with untrained surgeons. The operation time, accuracy, incidence of intraoperative errors, and postoperative complications were statistically better in the simulation-trained group in comparison with the conventional-trained group.

**Conclusion:**

Simulation provides a safe, effective, and ethical way for residents to acquire surgical skills before entering the operating room.

## 1. Introduction

Many factors affect the postgraduate surgical training including changes in working hours. Since 2009, the European Working Time Directive has reduced the maximum amount of hours spent working to an average of 48 hours per week [1]. Consequently, trainees should reach the same level of competency as their predecessors, when working hours are reduced [2]. Furthermore, it has been calculated that teaching surgical residents in the operating room costs $53 million per annum in the USA, which in turn causes further financial pressures in training procedure [3].

According to recent epidemiological studies, medical errors are considered as common causes of death in the USA [4]. The fact that patients are exposed to inexperienced healthcare practitioners has led the educational system to reevaluate medical training [5].

Simulation is a new developed educational procedure, in which trainees are able to improve their medical skills. This educational approach provides opportunities for repeated and safe medical training in a simulated environment [6]. Virtual reality, animal models, simulated patients, and static or interactive manikins are utilized effectively in simulation programs [7, 8].

Simulation has been supported by the Food and Drug Administration (FDA) as a fundamental part of surgical training in carotid artery stenting [9]. Similarly, the Residency Review Committee and the American College of Surgeons (ACS) have emphasized the importance of its role [10]. According to the American Board of Surgery, individuals are required to complete successfully an educational program entitled “The Fundamentals of Laparoscopic Surgery” (FLS) developed by the Society of American Gastrointestinal and Endoscopic Surgeons (SAGES), to be board certified in general surgery [11, 12].

More than 20 types of computer-based trainers have been developed for a wide spectrum of surgical interventions [13–15]. Simulation techniques can be applied in many health care domains such as surgery, obstetrics, invasive cardiology, anesthesia, critical care, and emergency medicine [16]. The use of simulation has been increased in the last few years, but it has not reached a widespread adoption in healthcare education and training. Although results are positive and encouraging, integration of simulation-based training into residents' curricula remains compromised [17], and simulation laboratories continue to be underutilized [18].

The aim of this systematic review is to determine whether surgical skills acquired through simulation-based training are transferable to the operating room.

## 2. Material and Methods

**Figure 1:**
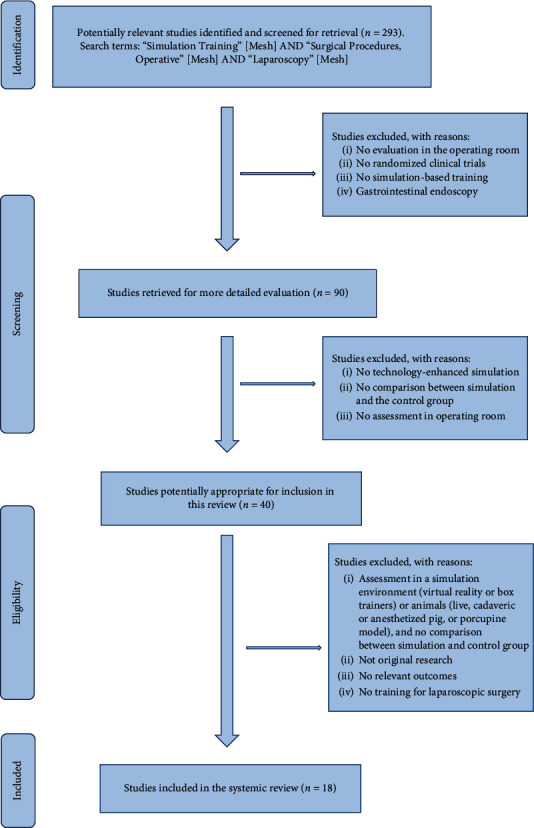
Trial flow. Flow chart showing selection of articles for review.

This review focused on published literature, which evaluates the transferability of simulation-based training into the real operating room. The literature search was carried out in the following databases: Embase, PubMed, the Cochrane Library and Current Contents, Clinical Trials Database (US), NHS Centre for Research and Dissemination Databases (UK), and National Research Register (UK). Articles published in English or German were included in our survey. The search strategy included the following keywords: “Simulation Training”[Mesh] AND “Surgical Procedures, Operative”[Mesh] AND “Laparoscopy”[Mesh].  Figure 1 demonstrates the elimination of articles that came up during the search process.

### 2.1. Inclusion and Exclusion Criteria

Randomized clinical trials (RCTs), which investigated the transferability of surgical skills in the real operating environment, were included in this review. Included studies compared the use of technology-enhanced simulation at any year of residency with conventional training activity. In all surveys, simulation was used as the educational procedure for teaching laparoscopic skills. Simulators included in this review were virtual reality, box trainers, task trainers, and cadaveric or live porcine models. The transferability of surgical skills was assessed in the real operating theater. Studies that evaluated performance in a simulation environment (virtual reality or box trainers) or animals (live, cadaveric, or anesthetized pig or porcine model) were excluded. We did not make exclusions based on the outcome or year of publication. Studies dealing with other minimally invasive techniques such as arthroscopy and gastrointestinal, airway, or urogenital endoscopy were excluded. Single-group pretest-posttest studies, nonrandomized studies, reviews, or meta-analyses were excluded.

### 2.2. Data Extraction and Analysis

An adopted coding framework based on PRISMA [19] and Cochrane handbook [20] was used to review the literature. Study quality was assessed according to the methods given in §6 of the Cochrane Reviewers' Handbook on a number of parameters, including quality of the reporting study methodology, methods of randomization and allocation concealment, blinding of trainers and outcomes assessors, and sample sizes. After identifying all potentially relevant studies of simulation-based training for health professions, two reviewers have screened all titles and abstracts (or full text) for inclusion.

### 2.3. Objectives and Outcome Measures

Objective of the study is to evaluate the effectiveness of simulation training and comparing this training modality with traditional education. Laparoscopic skills, surgical performance, and types of intraoperative errors will be compared between the two groups.

The following parameters will be assessed between participants in both modalities:

- Operative time,
- Intraoperative and postoperative complications,
- Laparoscopic skills,
- Camera navigation,
- Percentage of errors and economy of movements,
- Visuospatial ability,
- And technical proficiency.

## 3. Results

A total of 18 studies met our inclusion criteria. All after training assessments were conducted in the real operating theater, and all studies were RCTs (Table 1).

Two RCTs evaluated the role of simulation in inguinal hernia repair. Zendejas et al. [21] concluded that simulation-based education is associated with decreased operative time and improved surgical performance compared with the control group. Furthermore, reduced frequency of intraoperative and postoperative complications as well as shorter length of hospital stay were reported. Similarly, Kurashima and colleagues [38] reported statistically improved technical skills in the simulation group.

Camera navigation was assessed in two RCTs. Nilsson et al. compared simulation training with traditional training during a laparoscopic cholecystectomy [22]. Simulation-trained novice surgeons showed improved technical skills in simulation environment where the laparoscopic tasks were conducted faster than in the control group. However, in the operative theater, statistically significant differences regarding camera navigation were not detected between the groups. In accordance with these results, Franzeck et al. found no significant difference in camera navigation skills between simulation-trained participants and novice surgeons trained in an operating theater [23].

Six studies evaluated the transferability of simulation training during a laparoscopic cholecystectomy. In a RCT by Seymour et al. [24], participants were divided into a virtual reality group and a control group. In a real operating theater, gallbladder dissection was 29% faster in the simulation group, whereas increased risk of gallbladder injury or nontarget tissue burns were reported in the control group. These findings were confirmed in a study by Grantcharov et al. [25] where the laparoscopic-trained group performed the cholecystectomy significantly faster than the control group. Furthermore, percentage of errors and economy of movements were significantly improved after virtual reality training.

Palter et al. [26] demonstrated a curriculum to improve laparoscopic skills in a simulation environment, which includes case-based learning, virtual reality, box training simulation, and participation in operations. Participants following this curriculum showed statistically enhanced surgical skills in laparoscopic cholecystectomy compared with traditional-trained surgeons. In accordance, a RCT reported a statistically superior performance in the operating theater for participants following virtual reality simulation compared to the control group [27].

In a study by Ahlberg et al. [28], participants were trained in a virtual reality simulator until reaching a proficiency level. Then, surgeons in the simulation group and control group conducted their first ten laparoscopic cholecystectomies in real environment. Similarly to other studies, the performance of the control group was associated with an increasing number of errors. The surgical time in the control group was longer in comparison with the simulation group. Furthermore, the simulation group demonstrated a homogeneous surgical performance, whereas untrained surgeons showed considerable heterogeneity in the conducted procedure.

Box trainer was utilized in a survey conducted by Bansal et al. [29]. The laparoscopic training group showed statistically better results in the operative time (*p*=0.002), plane of dissection (*p*=0.002), and surgical performance according to the GOALS criteria.

Simulation was utilized for laparoscopic bilateral tubal ligation in two RCTs [30, 31]. Simulation-based education was found to be superior to traditional apprenticeship training regarding the performance of the procedure in the clinical setting. In both surveys, surgical skills were statistically more exceptional in the simulation group compared with the control group.

Two other RCTs described the role of simulation in performing laparoscopic salpingectomy [32, 33]. In both studies, transferability of surgical skills in the operating room was observed, whereas operative time was shorter for simulation-trained participants compared with conventionally trained surgeons. Furthermore, the simulation-trained group reached a level of operational performance, which was equivalent to an intermediately experienced gynecologist. In a survey conducted by Ahlborg et al. [34], residents followed virtual reality simulation training. Assessment was conducted during laparoscopic tubal occlusion. Visuospatial ability, flow score, and self-efficacy were significantly higher with the simulator-training groups in comparison with the control group. Duration of surgery was significantly shorter in the training groups.

Palter and Grantcharov [35] conducted a survey to compare traditional residency training with a comprehensive curriculum consisting of simulation-based and cognitive training. The transferability of surgical skills in the operating room was assessed during a laparoscopic colectomy. The curriculum group conducted this procedure with a statistically higher technical proficiency in comparison with the conventional group. Furthermore, simulation-trained residents performed more surgical steps of the procedure than the control group.

Orzech et al. [36] confirmed the transferability of simulation suturing skills in the operating room. Both virtual reality simulation and box trainer were effective. Simulation-trained surgeons conducted the procedure better compared to conventionally trained surgeons. In the control group, participants required six attempts intraoperatively to reach an acceptable result. This translates into increased operative time, hospital costs, and intraoperative complications. A survey by Van Sickle et al. [37] has demonstrated that a structured curriculum for laparoscopic suturing and knot tying is related to improved surgical skills in an operating room. Time, errors, and needle manipulation were found to be significantly fewer for the simulation-trained participants compared with the control group.

Table 1 describes the method of simulation used in the included surveys, number of participants, groups, type of after training assessment, and results.

## 4. Discussion

Utilization of simulation in residency programs has been increased in the last decade. Many surveys have been conducted to evaluate the transferability of surgical skills into the operating room. Our systematic review included RCTs, in which the assessment of different simulation methods occurred in the real operating theater. This review suggests that simulation-based training improves the operating performance among novice surgeons. Included studies evaluated the results of simulation during laparoscopic cholecystectomy, inguinal hernia repair, bilateral tubal ligation or occlusion, salpingectomy, colectomy, and laparoscopic suturing. There were significant variations in assessment methods, and studies have assessed different surgical skills and parameters. Despite these variations in assessments, all studies have reported the effectiveness of simulation training in clinical practice. Trained surgeons have performed the operations faster, with increased accuracy and low percentage of intraoperative errors or postoperative complications.

None of the studies have reported poorer performance in the simulation-trained group in comparison with the conventional-trained group. Simulation was associated with significantly fewer intraoperative errors and shorter operative time [21, 24, 25, 28, 29, 32, 37]. Three studies have evaluated the role of comprehensive curriculum in surgical training, reporting statistically better performance in this group [21, 26, 35]. The curricula consisted of cognitive training, simulation training, and participation in real operations. These results make the integration of simulation training into comprehensive curricula, a major challenge in residency education.

Transferability of laparoscopic skills was evaluated in a wide range of surgical procedures, and different methods of assessment were utilized. This heterogeneity confirms the beneficial role of simulation in many surgical specializations, such as general surgery, bariatric surgery, and gynecology. Most studies used virtual reality as the simulation method when a minority of them used box trainer, and only one study used porcine cadaver. The aim of our survey was not to compare different simulation methods. A RCT found no statistically significant differences between virtual reality simulators or box trainers [39]. According to this study, both procedures are equally effective educational simulators for novice surgeons.

To the best of our knowledge, this is the first systematic review that includes only RCTs. Other conducted reviews included randomized and nonrandomized trials, whereas the after training assessment occurred in a simulation environment, in porcine models or in an operating room [40–43]. The after training assessment in a simulation environment cannot be evaluated with high accuracy and the transferability of surgical skills in a real operating theater. Including only studies in which assessment was undergone during real operations, we have limited factors that could influence our results.

Our review and results are limited by the data provided in the included studies. A weakness of this review is that studies varied in terms of sample size, type of simulator, type of simulation-based training, assessment methods, and operative procedure. Different training curricula were used, and a considerable variability between studies in the length of training was documented. The duration of simulation-based training has been different among studies, making it difficult to recommend a specific standard training period before entering the operating room. Furthermore, the majority of studies have the potential for increased type I errors, since only simple statistical analyses were used.

## 5. Conclusion

This systemic review evaluated the transferability of surgical skills acquired through simulation-based training into the operating room. The included studies were RCTs and showed that simulation-based training leads to superior performance in the operative setting compared to conventional training. Therefore, simulation provides a safe, effective, and ethical way for residents to acquire surgical skills before entering the operating room.

## Figures and Tables

**Table 1:** Included randomized clinical trials which have evaluated the transferability of simulation-based training in a real operating room.

Study	Simulation method	Number of participants	Groups	Assessment	Results
Zendejas et al. [21]	Guildford MATTU TEP hernia task trainer (Limbs and Things, Ltd. Bristol, UK)	50 surgical surgeons	(i) Simulation-based mastery learning (ML) curriculum (ii) Standard practice (self-learning and intraoperative learning)	Totally extraperitoneal (TEP) inguinal hernia repair	Operative time was shorter, and operative performance (GOALS scale) was better in the simulation group (*p* < 0,05). Intraoperative and postoperative complications were statistically decreased for simulation-trained residents (*p* < 0,05).

Nilsson et al. [22]	LapSim virtual reality simulator (software version 2015, Surgical Science, Gothenburg, Sweden)	36 surgical novices without prior laparoscopic experience	(i) Camera group (ii) Simulation-based cholecystectomy (procedure group) (iii) Control group	Camera assessment during a laparoscopic cholecystectomy	No statistically significant differences in camera navigation skills were found during a laparoscopic cholecystectomy between the groups. On the simulation-based test (LASTT model), technical skills were significantly better for the camera and the procedure group compared with the control group.

Franzeck et al. [23]	LAP Mentor^TM^ (Simbionix USA, Cleveland, OH). ProMISTM surgical hybrid simulator (Haptica Ltd., Dublin, Ireland)	24 pregraduation medical students	(i) Simulation group (ii) Training in the operating room	Camera assessment test in the operating room	Both groups improved their navigation skills significantly. The simulation group showed a trend towards better performance.

Seymour et al.[24]	Minimally invasive surgical trainer-virtual reality (MIST-VR) system (Mentice AB, Gothenburg, Sweden)	16 surgical residents	(i) Virtual reality (ii) Control group	Laparoscopic cholecystectomy	Simulation group performed the procedure 29% faster. Intraoperative complications (gallbladder injury or burn of nontarget tissue) occurred more commonly in the control group (*p* < 0,039).

Grantcharov et al. [25]	MIST-VR system (Mentice AB, Gothenburg, Sweden)	16 surgical trainees	(i) Virtual reality (ii) Control group	Laparoscopic cholecystectomy	Participants in the simulation group conducted the surgery statistically faster (*p*=0,021). Percentage of errors and economy of movements were significantly improved after virtual reality training (*p*=0,003).

Palter et al. [26]	LapSim virtual reality simulator	20 general surgery residents(PGY 1-2)	(i) Structured training and assessment curriculum (STAC) group (ii) Conventional residency training	Laparoscopic cholecystectomy	Residents performed five sequential laparoscopic cholecystectomies in the operating room. The STAC group conducted the first four operations statistically better than the control group (OSAT global rating scale). In the fifth procedure, there was no significant difference. Participants in the STAC group showed improved nontechnical skills compared with the control group (*p*=0,027).

Palter and Grantcharov [27]	LapSim VR simulator (Gothenburg, Sweden, 2008 version)	16 surgery residents(PGY 1-2)	(i) Virtual reality group (ii) Conventional residency training group	Laparoscopic cholecystectomy	Individualized deliberate practice on simulator results in a statistically superior performance in the operating theater for the simulation group compared with the control group (*p*=0,003).

Ahlberg et al. [28]	LapSim	13 surgical residents	(i) Training group (ii) Control group	Laparoscopic cholecystectomy	Virtual reality group outperformed the control group in terms of operative time and number of errors intraoperatively.

Bansal et al. [29]	Box trainer, the Tubingen MIC-Trainer (Richard Wolf GmbH, Germany)	17 surgery residents	(i) Laparoscopic training group (ii) Standard training group	Laparoscopic cholecystectomy	The laparoscopic training group showed statistically better results in the operative time (*p*=0.002), plane of dissection (*p*=0.002), and GOALS criteria. The rate of gallbladder perforation was higher for untrained surgeons, but a statistically significant difference was not found.

Banks et al. [30]	Laparoscopy simulator (Limbs and Things, Bristol, UK) and an operative laparoscopy tower	20 residents(PGY 1)	(i) Simulation-based training and surgical training in the operating room (ii) Surgical training in the operating room	Laparoscopic bilateral tubal ligation	Simulation group performed the intervention statistically better than the control group. Surgical skills in simulation-trained residents were improved compared with the control group (*p* < 0,005).

Gala et al. [31]	Psychomotor board testing with a peg board test	44 lower-level residents (PGY 1-2) and 66 upper-level (PGY 3-4)	(i) Traditional training	Laparoscopic Pomeroy bilateral tubal ligation	Simulation-trained surgeons showed significantly higher normalized simulation scores (*p* < 0,01) and higher levels of competence on the simulated tasks (*p* < 0,01). Simulation group had improved surgical skills (Likert scale) in the operating theater compared with the control group (*p* < 0,03).

Larsen et al. [32]	LapSim Gyn v 3.0.1 (Surgical Science, Gothenburg, Sweden)	32 trainees in gynecological specialty(PGY 1 and 2)	(i) Intervention group (ii) Control group	Laparoscopic salpingectomy	Intervention group performed the surgery with statistically significant superiority compared with the control group (*p* < 0,001). Operative time was significantly shorter in the simulation group (*p* < 0,001).

Patel et al. [33]	Porcine cadaver	22 residents	(i) Simulation group (ii) Control group	Laparoscopic salpingectomy	Simulation can improve significantly surgical skills (OSAT scores) in laparoscopic salpingectomy. Combination of simulation and traditional training is recommended.

Ahlborg et al. [34]	LapSim Gyn VR simulator (Surgical Science, Gothenburg, Sweden)	28 trainees	(i) Simulator training (ii) Simulator training with mentorship (iii) Control group	Laparoscopic tubal occlusion	Visuospatial ability, flow score, and self-efficacy were significantly higher for both the simulator-training groups compared with the control group. Duration of surgery was significantly shorter in the training groups. Differences in surgical performance between the two simulation groups were not detected.

Palter et al. [35]	LapSim (Surgical Science, Gothenburg, Sweden)	25 surgical residents(PGY 2-4)	(i) Curriculum training group (ii) Conventional residency training	Laparoscopic right colectomy	Curriculum group showed statistically significant superiority in technical proficiency compared with the conventional group (OSATS score, *p* < 0,03). Curriculum-trained participants performed more operative steps than residents in the conventional group.

Orzech et al. [36]	LapSim	24 surgical residents(PGY 2 or above)	(i) Virtual reality (ii) Box trainer (iii) Conventional training (iv) Experienced surgeons	Laparoscopic suturing	No statistically significant differences were found between virtual reality and box trainer in time and technical proficiency. Box training is thought as a cost-effective training program, whereas virtual reality provides a time-efficient education. Simulation-trained surgeons conducted the procedure better compared to conventionally trained surgeons.

Van Sickle et al. [37]	Virtual reality and box trainer	22 surgery residents (PGY level 3, 5, or 6)	(i) Curriculum training group (ii) Standard training group	Laparoscopic intracorporeal suturing and knot tying during a laparoscopic Nissen fundoplication	Laparoscopic suturing training group performed the suturing task statistically faster with a reduced rate of errors and fewer needle manipulations than the control group (*p* < 0,003 and *p* < 0,01, respectively).

## Data Availability

The data used to support this study can be accessed in PubMed. The data and published articles that support the conclusions are reported in references.

## Abbreviation

RCT: Randomized clinical trial.
